# Association of maternal ABO blood type with lesion level and birthweight of children with spina bifida: a descriptive study

**DOI:** 10.25122/jml-2024-0072

**Published:** 2024-05

**Authors:** Thomas Lee Farley

**Affiliations:** 1 Arkansas Spinal Cord Commission, Department of Health, Little Rock, AR, USA

**Keywords:** spina bifida, neural tube defects, blood type, lesion level, birthweight, hydrocephalus, folic acid

## Abstract

The etiology of spina bifida, a neural tube birth defect, is largely unknown, but a majority of cases are thought to be genetic in origin. Although maternal blood type was found not to be associated with the occurrence of spina bifida, the analysis was never extended to other aspects of the disorder. The purpose of this descriptive study was to determine if maternal blood type was related to characteristics of children with spina bifida. The blood type of 221 mothers of children with spina bifida enrolled on the Arkansas Spinal Cord Disability Registry from 1995 to 2008 was obtained by mailed questionnaire. All children were community-dwelling and from singleton pregnancies. As expected, analysis of mother-child data showed that the distribution of mothers’ blood type was not statistically different from the general population (chi-squared, *P* = 0.9203). However, the blood type of these mothers was associated with their child’s lesion level (chi-squared, *P* = 0.011). Mothers with blood type A more frequently had children with thoracic lesions; mothers with non-A blood types more frequently had children with lumbar and sacral lesions. In addition, mean birthweight differed by mothers’ blood type (analysis of variance, *P* = 0.025). Children of mothers with blood type A had the highest mean birthweight, while those of mothers with blood type AB had the lowest. Also, hydrocephalus was present more frequently in children with thoracic lesions compared to those with lumbar and sacral lesions (chi-squared, *P* = 0.001). Interestingly, these results were significant for female children but not for male children. In conclusion, maternal blood type was associated with lesion level and birthweight of children with spina bifida.

## INTRODUCTION

Neural tube defects (NTDs) are birth defects affecting the brain and spine of the developing fetus. Conditions affecting the brain include anencephaly, encephalocele, craniorachischisis, and iniencephaly. Conditions affecting the spinal cord are spina bifida and tethered cord [1]. Spina bifida is the most common NTD, identified by a lesion of the cervical, thoracic, lumbar, or sacral area of the vertebral column. Cervical lesions are usually not survivable; thoracic, lumbar, and sacral lesions are survivable, with higher (rostral) lesions associated with greater loss of body function.

At birth, four characteristics distinguish children with spina bifida from the general population: sex, lesions, birthweight, and hydrocephalus. The majority of children born with spina bifida are female. Although in the United States and the state of Arkansas, the live birth sex ratio is essentially equal or slightly favoring males in the general population [2], the preponderance of children born with spina bifida is female [3,4].

Children with spina bifida have spinal cord lesions that vary in severity and level on the vertebral column. The more the spinal cord is exposed to cerebrospinal fluid or is outside the column, the greater the probability of impairment. Likewise, higher lesions affect the nerves of more organs and result in greater body impairment.

On average, children with spina bifida have a lower birthweight than comparable children in the general population [5].

The majority of children with spina bifida have hydrocephalus at birth or will develop it during their lifetime [6]. Hydrocephalus arises from distortion of the nervous system and tissue dislocation as a consequence of the spinal defect. As a result, the circulation of the cerebrospinal fluid is obstructed and accumulates in the cranium, while the spinal cord may receive an insufficient amount [7]. This associative disorder results in the abnormal build-up of cerebrospinal fluid and pressure in the brain, resulting in loss of function, damage, or death if untreated. A shunt is installed to drain excess fluid to a location where the body can safely absorb it.

Although a large part of the etiology of NTD and spina bifida is unknown, there is agreement that it is complex and consists of environmental and genetic factors [8]. On the environmental side, certain teratogens [9] and a diet insufficient in folate [10] have been linked to spina bifida. After folic acid fortification of the United States grain supply in 1998, spina bifida prevalence fell by 23%, with most of the decrease occurring among upper lesions while lower lesions remained relatively stable [11,12]. On the genetic side, certain chromosome and single-gene syndromes [9,10] have been linked to spinal bifida. An estimated 60–70% of cases are thought to be genetic in origin [10], and although evidence from families with a history of NTD points to a major gene, none has been identified [9]. In addition, research has shown that upper lesions occur during neurulation or closing of the neural tube, whereas lower lesions arise from the later process of canalization [9]. Therefore, thoracic lesions are thought to be caused by an etiology separate from lumbar and sacral lesions [13], but no associated gene or other variable has been reported. Thus, the vast majority of all NTDs remain unexplained [10].

Early research compared ABO and Rh blood types of mothers with a history of spina bifida to various samples of mothers from the general population without such a history. Rh-negative blood type findings were controversial [14], and no relationship was found for ABO blood type [14-17]. Following these negative findings, there is no mention of maternal or population blood type as a research parameter in the NTD literature. Consequently, there is no evidence that maternal blood type is not a risk factor within the NTD population. The present study is an effort to close this research gap. For that reason, this descriptive study investigated the relationship between mothers’ ABO and Rh blood types and characteristics of children with spina bifida, including sex, lesion level, birthweight, and presence of shunt.

## MATERIAL AND METHODS

Data for the present study were collected in 1995–1996 and 2008 by the Arkansas Spinal Cord Commission (ASCC). ASCC, a division of the Department of Health, is mandated by law to maintain a registry to provide services to all residents with spinal cord disability, including individuals with spina bifida. Study participants were drawn retrospectively from the registry and consisted of 221 mother-child pairs. All children were community-dwelling and were from singleton pregnancies. Arkansas residents with spinal cord damage were admitted to the registry if they met three of four criteria documented by a physician: lack of normal motor control, lack of normal sensation, loss of normal bladder control, and loss of normal bowel control. Newborn children with spina bifida met three or all four of the criteria. Physician documentation for a child with spina bifida included diagnosis, other abnormalities, and the functional level of the lesion that became part of ASCC’s medical record. For neural tube defects, ASCC criteria exclude all head trauma, abortions, and stillbirths. Spina bifida occulta is usually excluded because the severity of disability does not meet the eligibility criteria. The registry does include myelomeningocele, meningocele, and spina bifida – not specified. Children included in the study had these types of spina bifida.

No mother-child with a history of in-utero spina bifida repair was enrolled in the registry during the study period. Children with such a history would have been excluded from the study because of post-surgery changes in functional level and decreased need for shunts [18].

Subjects were identified through two different postal surveys. The first survey was conducted between 1995 and 1996 and collected information about the mothers’ reproductive and disease history. The methodology and findings of the 1995–1996 survey have previously been reported [4]. Of the 271 mothers who participated, 152 knew their blood type and were included as subjects for the present study. The second survey of mothers was conducted in 2008; it focused on collecting the mothers’ blood type and disease history and was less comprehensive than the 1995–1996 survey. The second survey was mailed to all 455 mothers whose child was listed on the registry in 2008. A total of 69 additional, non-duplicated mothers were identified from children admitted between 1996 and 2008 and from mothers who had not responded previously, for an estimated response rate of 37.5%. Thus, the total number of subjects for the present study was 221: 152 from the 1995–1996 survey and 69 from the 2008 survey.

Responses to both surveys were self-reported. All mothers, regardless of child age, were mailed a survey. Questionnaires with incomplete responses were followed up with telephone calls from study staff. Mothers reported their child’s lesion level by vertebra location. Lesion levels were grouped into three regions: thoracic, lumbar, and sacral levels. Thoracic lesions were defined as upper lesions; lumbar and sacral levels were defined as lower lesions. If the mother did not know the child’s lesion level (12 cases), the level from the referring physician’s report in the medical record was used. For functional levels involving a range, the highest level in the range was used. To verify the accuracy of the reported lesion level, the medical record was compared to a postal zip code stratified sample of 40 cases out of 209 cases where a level was reported. The medical record and reported functional level matched 90% (36/40) of the sample cases; there was 100% agreement for all 40 cases regarding regional and upper/lower groups.

Maternal blood type was reported using the ABO and Rh classifications. During analysis, it became evident that an A vs. non-A categorization was statistically more powerful than the A, B, AB, and O categorization. Therefore, non-A blood type was defined as types B, AB, and O. Child birthweight was reported in pounds and ounces and converted to g. Low birthweight was defined as less than 2,500 g. Significance tests used chi-squared, odds ratio, *t*-test, and analysis of variance (ANOVA), with a *P* value of <0.05 considered significant. The Shapiro–Wilk test, as well as multivariable logistic and linear regression, were used. Covariates considered in the regression models included maternal race, child’s sex, and lesion level. Covariates remained in the model when a 10% or greater effect was observed in the results. Wizard 2 was used for the data analysis. The ASCC financed both surveys.

## RESULTS

### Demographics

**Figure 1:**
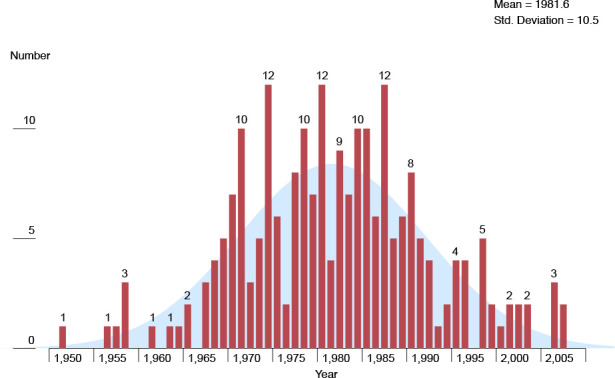
Distribution of year of child birth

The average age of mothers at birth was 25.5 years, and the mean weight of children was 3,148 g (Table 1). The mother’s blood type was well distributed across the ABO range. In total, 57.9% of the children were female. The distribution of child lesion levels was thoracic (41.2%), lumbar (52.0%), and sacral (6.8%); there were no cervical lesions. Thus, by definition, upper lesions represented 41.2% and lower lesions 58.8%. Rh blood type was positive for 86.9% of the mothers. Nearly 80 percent (79.6%) of the children had a shunt installed, indicating the presence of hydrocephalus. The year of childbirth ranged from 1951 to 2007, with a mean of 1981.6 and a (tied) mode of 1974, 1981, and 1987 (Figure 1). More than 90 percent (91.4%) of the children were born before mandatory fortification of grains went into effect on 1 January 1998.

**Table 1:** Selected characteristics of the participants

Characteristic	No.	Percent
**For mothers**		
Age at child’s birth, years, mean ± s.d.	25.5 ± 5.6	
Race		
White	197	89.1%
African-American	19	8.6%
Asian/Pacific Islander/Other	5	2.3%
ABO blood type		
A	78	35.3%
B	29	13.1%
AB	18	8.2%
O	96	43.4%
Rh blood type		
Positive	192	86.9%
Negative	29	13.1%
**For children**		
Weight at birth, g, mean ± s.d.	3,149 ± 570.7	
Sex		
Male	93	42.1%
Female	128	57.9%
Lesion level		
Thoracic	91	41.2%
Lumbar	115	52.0%
Sacral	15	6.8%
Shunted hydrocephalus		
Yes	176	79.6%
No	45	20.4%
Year of birth, mean ± s.d.	1981.6 ± 10.5	
Birth before/after 1 January 1998^†^		
Before	202	91.4%
After	19	8.6%

† Date of mandatory folic acid grain fortification in the United States

### Representativeness of the subjects

Children and their mothers included in the study were compared with all children on the registry to determine their similarity. In the 2008 survey, mothers included in the study (*n* = 221) represented 48.6% of the 455 mothers of children with spina bifida on the registry. The distribution of birth years did not depart from a normal distribution (Shapiro–Wilk, *P* = 0.317; Figure 1). Children included in the study from 148 residential zip codes represented a geographical area of 75.9% of the 195 zip codes for children on the registry with spina bifida. In total, 89.1% of the mothers included in the study were white, compared to 82.4% of registry enrollees. Of children included in the study, 42.1% were male, and 12.2% lived in Pulaski County, compared to 48.4% and 15.2%, respectively, for registry enrollees. Therefore, with nearly half of the mothers and their children on the registry in 2008 enrolled in the study, comparable values for the above traits, and a normally distributed sample for the year of childbirth, study participants were deemed representative of all children with spina bifida on the registry and their mothers.

### Mother’s blood type

In 2021, the ABO blood type distribution in the United States was A, 40%; B, 11%; AB, 4%; and O, 45%. Rh blood type was positive in 84% and negative in 16% of cases [19]. The blood type of mothers included in the study was not significantly associated with the United States ABO distribution (chi-squared 0.01, df1, *P* = 0.9203) or the Rh distribution (chi-squared 0.33, df1, *P* = 0.5608). Moreover, maternal blood type was not significantly related to the child’s sex (Table 2). However, every blood type had a preponderance of female sex, with type O being the highest at 62.5% (Table 3). Female preponderance differed by lesion level: mothers with blood types A and O had female children with upper lesions more frequently, whereas mothers with non-A blood types more frequently had female children with lower lesions.

**Table 2:** Child sex and maternal ABO blood type

Blood type	Child sex		Chi-squared *P* value	Odds ratio	Odds ratio 95% CI
	Male	Female			
A	38	40	0.509		
B	12	17			
AB	7	11			
O	36	60			
A	38	40		1.52	0.8705–2.654
Non-A^†^	55	88			

† Non-A consists of blood types B, AB, and O

**Table 3:** Child sex distribution for maternal ABO blood type and lesion level

Blood type	Upper lesion		Lower lesion		Totals		% Female^*^
	Male	Female	Male	Female	Male	Female	
A	19	24	19	16	38	40	51.3%
B	4	3	8	14	12	17	58.6%
AB	3	4	4	7	7	11	61.1%
O	12	22	24	38	36	60	62.5%
Totals	38	53	55	75	93	128	
% Female^†^	58.2%		57.7%				

* Percents calculated on blood type total counts.

† Percents calculated on lesion total counts.

### Child’s lesion level

Maternal ABO blood type was associated with child lesion level (*P* = 0.011; Table 4). Mothers with non-A blood had 2.4 times greater odds of having a child with a lower lesion than mothers with type A blood (OR = 2.43; 95% confidence interval (CI), 1.38–4.27). The blood type/lesion level association differed by the child’s sex. Mothers with non-A type blood had three times greater odds of having a female child with a lower lesion than mothers with type A blood (OR = 3.05; 95% CI, 1.40–6.61). Male sex was not significantly associated with lesion level.

**Table 4:** Lesion level and maternal ABO blood type for all children and child sex

Group	Blood type	Child lesion level		Chi-squared *P* value	Odds ratio	Odds ratio 95% CI
		Upper	Lower			
All children	A	43	35	0.011		
	B	7	22			
	AB	7	11			
	O	34	62			
					
	A	43	35		2.4315	1.3816–4.2795
	Non-A^†^	48	95			
**Child sex**						
Male	A	19	19		1.8947	0.8143–4.409
	Non-A	19	36			
Female	A	24	16		3.0517	1.4086–6.6117
	Non-A	29	59			

† Non-A consists of blood types B, AB, and O

Logistic regression was used to analyze child lesion levels. It was found that mothers with blood type B had 3.9 times greater odds than mothers with blood type A to have a child with a lower lesion (OR = 3.9; 95% CI, 1.4–10.0). Mothers with blood type O had 2.2 times greater odds than mothers with blood type A to have a child with a lower lesion (OR = 2.2; 95% CI, 1.2–4.1). In total, 58.2% of children with upper lesions and 57.7% with lower lesions were female (Table 3). White mothers gave birth to 93.4% of children with upper lesions and 86.2% of children with lower lesions (results not shown).

### Child’s birthweight

The birthweight was known for 218 of the 221 children. Maternal blood type was significantly associated with child birthweight (*P* = 0.026; Table 5), which was the highest among mothers with blood type A (3,260 g) and the lowest among mothers with blood type AB (2,870 g). Mothers with blood type A had children with significantly higher birth weights than mothers with non-A blood types (*P* = 0.003). The association between blood type and birthweight differed by the child’s sex and lesion level. The birth weights of female children born to mothers with A and non-A blood types were significantly different (*P* = 0.001), whereas those of male children were not (*P* = 0.458). The birth weights of children with lower lesions born to mothers with A and non-A blood were significantly different (*P* = 0.023), whereas those of children with upper lesions were not (*P* = 0.202). The presence of a shunt was a significant variable for the birthweight analysis only for female children: female children with a shunt (*n* = 100) had a mean weight of 3,206 g vs. female children without a shunt (*n* = 28) with a mean weight of 2,907 g (*t*-test, *P* = 0.012). The presence of shunt was not a significant variable for all children, male children, upper/lower lesion level, and A/non-A blood type for the birthweight analysis (results not shown).

**Table 5:** Child birthweight and maternal ABO blood type for selected groups

Group	Blood type	No.	Mean birthweight (g)	ANOVA *P* value	*t*-test *P* value
All children	A	77	3,260	0.026	
	B	28	2,894		
	AB	17	2,870		
	O	96	3,090		
				
	A	77	3,260		0.003
	Non-A^†^	141	3,022		
**Child sex**					
Male	A	38	3,217		0.458
	Non-A	54	3,124		
Female	A	39	3,385		<0.001
	Non-A	87	3,029		
Child lesion level					
Upper	A	42	3,337		0.202
	Non-A	48	3,189		
Lower	A	35	3,260		0.023
	Non-A	93	3,002		

† Non-A consists of blood types B, AB, and O

Linear multiple regression was used to analyze child birth weight. The results of the regression indicated that maternal race and A/non-A blood type explained 8.3% of the variance (*R^2^* = 0.091; *F*(2,215) = 10.803; *P* < 0.001).

The mean birthweight of children included in the study was 3,162 g for male children and 3,139 g for female children. In 2008, the mean birthweight of Arkansas singleton children was 3,306 g for male children and 3,184 g for female children [2]. Thus, male and female children included in the study had a mean birthweight of 144 and 45 g less, respectively, than the general population. Children included in the study born to white mothers had a higher mean weight (3,194 g) than those born to non-white mothers (2,726 g) (*P* < 0.001). In 2008, the mean birthweight of Arkansas singleton children was 3,301 g for whites and 3,052 g for non-whites [2]. Thus, white and non-white children included in the study had a mean birthweight of 107 and 326 g less, respectively, than the general population.

Children with upper lesions had a higher mean weight (3,258 g) than those with lower lesions (3,072 g) (*P* = 0.018).

Of the 218 children with a known birthweight, 29 (13.3%) were below 2,500 g. Low birth weight was not significantly associated with mother’s ABO or Rh blood type, mother’s race, child’s sex, lesion level, or presence of shunt (results not shown). Shunted hydrocephalus was associated with the child’s lesion level (*P* = 0.001; Table 6). The association between lesion level and hydrocephalus differed by sex, hydrocephalus being significantly more frequent among female children (*P* < 0.001) but not among male children (*P* = 0.222). Hydrocephalus was not significantly associated with the mother’s blood type, mother’s race, child’s sex, or birth weight (results not shown). A shunt was installed for 87.9% of children with thoracic, 78.3% of children with lumbar, and 40.0% of children with sacral level lesions.

**Table 6:** Hydrocephalus for lesion level for all children and child sex

Group	Lesion level	Hydrocephalus		Chi-squared *P* value
		No	Yes	
All children	Thoracic	11	80	<0.001
	Lumbar	25	90	
	Sacral	9	6	
**Child sex**				
Male	Thoracic	4	34	0.222
	Lumbar	11	38	
	Sacral	2	4	
Female	Thoracic	7	46	<0.001
	Lumbar	14	52	
	Sacral	7	2	

### Rh blood type

Rh blood type was not significantly associated with mother’s ABO blood type, mother’s race, child’s sex, birthweight, lesion level, or hydrocephalus (results not shown).

### Folic acid fortification

There were not enough children born after 1 January 1998 to allow an analysis before and after grain fortification.

## DISCUSSION

This study has shown mothers’ ABO blood type to be statistically associated with the lesion level and birthweight of their children with spina bifida. Results were split along two dimensions: the mother’s A/non-A blood type and the child’s sex. Also, a preponderance of female children was found for all mother’s ABO blood types. In addition, hydrocephalus was found to be associated with lesion level and was also significant for female children but not male children. The distribution of ABO and Rh blood types of mothers included in the study was not statistically different from the general population and was consistent with the findings of previous studies [14–17].

A variety of diseases and disorders have been linked to ABO blood types [20]. Although controversial, with few mechanisms identified, ABO blood type-related findings may provide insight into disease susceptibility and offer new clues for research. In particular, Kumar *et al*. found that individuals with blood type A (A and AB) were more susceptible to enterotoxigenic *Escherichia coli* infections than individuals with non-A (B and O) blood type, resulting in more severe diarrhea [21]. They demonstrated that enterotoxigenic *Escherichia coli* agglutinated red blood cells and intestinal epithelial cells of individuals with blood type A but rarely those of individuals with blood type non-A. A similar mechanism may be present in mothers of children with spina bifida. The finding that 79.6% of children had a shunt installed is consistent with the results of Kim *et al*., who reported that 79.9% of 3,558 patients had undergone at least one hydrocephalus-related operation [22]. The association between hydrocephalus and lesion level revealed in the present study agrees with the results of Verhoef *et al*., who reported a greater percentage of hydrocephalus in upper lesions of patients with spina bifida [6]. Historically, spina bifida cases have been categorized into two groups based on the location of the spinal cord lesion. Although upper and lower lesions are considered heterogeneous, no maternal trait associated with child lesion level has been identified. Maternal ABO blood type, as this study has shown, was associated with the child lesion level and offers genetically based evidence for the heterogeneity of upper and lower lesions. ABO blood type is determined by an autosomal co-dominant gene [23]. The significant results obtained in the case of female but not male children regarding the association between the mother’s blood type, lesion level, and birthweight appear consistent with a sex-influenced trait as hypothesized by Byrne *et al*. [24]. Deak *et al*. [25] elaborated that a single, dominant gene with reduced penetrance may be responsible for the maternal and sex-influenced effects documented in families with a history of NTDs.

Both supplementation and fortification of the diet with folic acid have reduced the prevalence of NTDs [12,26]; however, the preponderance of female children [27] and the overall severity [12] of the children was also reduced. The mechanism by which folic acid has contributed to these changes is unknown, adding to the etiologic complexity of the disorder. This study’s findings may offer some insight into this question. Using Table 3 as an example, how can the numbers of this ‘essentially’ pre-fortification sample be adjusted to reflect a post-fortification sample in which overall prevalence and severity are reduced and the number of male and female children are similar? The most direct way is to lower the number of the 128 female children to match that of the 93 male children. This would decrease overall prevalence by 15.8% and eliminate the preponderance of female children. For upper lesions, a similar decrease would reduce overall severity. Hence, ‘excessive numbers of female children’ may have benefited the most from dietary folic acid programs. The preponderance of female children greatly contributes to this study’s significant results. Replication of this study in a folate-fortified population in which the preponderance of female children has been reduced may not yield similar results. Because of the greater reduction of upper lesions following folic acid fortification, these lesions have been described as ‘folate sensitive’ [12]. The present study involved a pre-fortification sample and found maternal A/non-A blood type significantly associated with overall lesion level and female sex. Differences in the distribution of lesions by level and sex might depend on the sensitivity of the mother’s blood type to folate. Laboratory analysis should be able to determine if such sensitivity exists and its relative strength. No comparable literature reporting on the mother’s blood type and the characteristics of children with spina bifida has been found. Only one study, by Beyazit *et al*., reported child birthweight and mother’s blood type for 2,177 mothers from the general population in Turkey, with the following results: type B, 3,005 g; AB, 3,127 g; A, 3,137 g; and O, 3,294 g (statistical test not specified, *P* <0.001) [28]. Blood types AB and B share the lowest birthweights in both this study and the one conducted in Turkey, but the positions of the other two blood types, A and O, were reversed. Also, the authors reported no apparent difference in child birthweight between mothers with blood type A or non-A. Thus, based on this one report, it appears that our results regarding birthweight are not reflective of the general population but are unique to the mothers included in this study.

This study’s finding that male and female children with spina bifida had lower mean birthweights than the general population is consistent with the results of Wald *et al*., who reported singleton male and female infants with spina bifida were 200 and 240 grams less, respectively, than singleton controls [5].

### Study limitations

The study has several limitations. Data regarding mothers’ blood type was collected during the 1995–1996 survey investigating the reproductive history of the mother. As a result, several key variables regarding the mother and child with spina bifida were not optimal for the present study or were not available. For example, maternal blood type determined by laboratory analysis would have been preferable to self-reporting. In addition, the blood type of the biological father and all siblings would have added much to the study’s findings. Additional covariates, such as maternal height and pre-pregnancy weight, may have improved the child birthweight regression analysis. Also, the lack of general population baseline data regarding child birthweight and mother’s blood type limited the birthweight analysis. Child lesion level, as reported in the medical record, would also have been preferable to self-reporting. A larger number of participants, in particular, more non-whites, would have resulted in a more robust analysis and interpretation. Although the subjects were drawn from the same geographical area, they were selected 13 years apart. The 2008 survey increased the number of participants, but a single data collection point would have avoided any population changes between the surveys. Although study variables were well defined and kept to a minimum, it is possible that these findings were due to chance. Besides, no study variable or scientific explanation pointed to a plausible biological mechanism other than the one provided by Kumar *et al*. [21]. The study’s findings were not random; they were patterned and consistent with other spina bifida research. However, the findings may have been skewed due to bias in the ascertainment of study participants. Mothers were invited to participate if their child was alive in the survey year. Mothers whose children had previously died were not invited. Thus, only surviving children and their mothers were asked to participate. The preponderance of female children in this study may be caused by a greater loss of male children any time before the survey year. Mai *et al*. reported a total loss of 29.7% of 2,593 spina bifida-related pregnancies in the years before and after folic acid fortification due to terminations (*n* = 355), stillbirths (*n* = 156), and infant deaths (*n* = 258) [12]. Therefore, it is possible that more female children were surveyed because they outlived the male children.

## CONCLUSION

If this study’s findings regarding maternal blood type are confirmed, a wide variety of questions and opportunities would open up for future researchers. It may be easy to survey mothers from existing associations, but more efficacious studies will require control of multiple variables and a sample population of sufficient size. Three methodologies are offered for consideration: 1) Study all mothers with spina bifida-related pregnancies that were terminated, stillborn, or died before age 1 from the same geographical area, both pre- and post-folic acid fortification. This approach would provide excellent confirmation data and help answer the long-standing question of whether spina bifida affects both sexes equally and whether more male children die before female children (pre-fortification) or female children are more susceptible. Hospitals have the resources for blood typing and most likely already have some of this data on file. However, birth defect registries and other programs could gain access to this information. Another approach would be to add maternal blood type to an existing, ongoing database or the database of a recently completed study of children with spina bifida. 2) Draw blood samples from mothers who have recently experienced a spina bifida-related pregnancy along with matched controls and examine them in the laboratory. 3) Conduct a follow-up study of Arkansas children with spina bifida and their mothers to note changes in lesion level, birthweight, and maternal blood type since 2008. In conclusion, maternal ABO blood type was found to be associated with lesion level and birthweight of the child with spina bifida and provides genetically based evidence for the heterogeneity of upper and lower lesions.

## Personal thanks

The author acknowledges the Spina Bifida Support Group of Arkansas for their endorsement.
